# Commentary: Functional Connectivity in the Left Dorsal Stream Facilitates Simultaneous Language Translation: An EEG Study

**DOI:** 10.3389/fnhum.2017.00064

**Published:** 2017-02-14

**Authors:** Roman Koshkin, Alex Ossadtchi

**Affiliations:** ^1^Center for Cognition and Decision Making, National Research University Higher School of EconomicsMoscow, Russia; ^2^Laboratory of Control of Complex Systems, Institute of Problems of Mechanical Engineering, Russian Academy of SciencesSt. Petersburg, Russia

**Keywords:** simultaneous interpreters, EEG, functional connectivity, dorsal stream, lexical decision task

Simultaneous language interpreting (SLI) places extreme requirements on the cognitive control skills (Yudes et al., 2011; Hervais-Adelman et al., 2015) acquired by multilinguals through intensive training and practice (Chernov, 2004). Not only does SLI involve constant concurrent retrieval of words and collocations in the source and target languages, it also requires their retention and manipulation in working memory, tracking both source and target discourse as well as performing repeated language switching (Moser, 1978; Moser-Mercer et al., 2000; Christoffels et al., 2006).

Previous research has shown that systematic SLI practice causes structural neuroplasticity in simultaneous interpreters (SIs). Specifically, Elmer et al. (2014) provided evidence for reduced gray matter volumes in SIs compared to multilingual controls. To further explore neuroplasticity in SIs, Elmer and Kühnis (2016) predicted the existence of functional cortical connectivity changes induced by exposure to SLI.

Based on the dual-stream model of speech processing (cf. Hickok, 2012), and the assumption that SLI practice places high demands on sensory-to-articulation mapping, Elmer and Kühnis (2016) analyzed two preselected regions of interest (ROIs) within the dorsal stream, namely BA 41/42 (auditory-related cortex), and BA 44/45 (Broca's area), expecting them to be more functionally coupled in SIs than in multilingual control subjects.

In the experiment, 12 professional SIs and the same number of multilinguals with no SLI experience were tested in a mixed and unmixed auditory semantic decision task which consisted in judging word congruency by pressing one of the buttons. In the unmixed condition the words were delivered sequentially in the same language (German or English), while in the mixed condition the languages were different.

Consistent with the prediction, analysis of EEG data revealed stronger functional coupling in the theta band (~4–7 Hz) between the ROIs in SIs than in controls. This result is very interesting because it suggests that SLI proficiency may be a matter of not only structural, but also functional changes in the brain.

In this commentary we offer several methodological considerations. First, the experimental task only included English and German words, and other language combinations may have produced a different pattern of results. For example, the authors could have included another experimental condition with French and English word pairs. This would have required almost no effort in recruiting extra participants: the autobiographical data show that all of the SIs rated their proficiency in French at least 3 on a scale of 1–6, while 10 out of 12 controls also knew French. Obtaining similar results in that condition would have made the authors' case stronger.

Another potential issue may be the unbalanced gender representation within groups: 10 out of 12 participants were female in each group. A number of authors (cf. Ponton, 1987; Hampson and Kimura, 1988; Gran and Dodds, 1989; Fabbro, 1992; Ojemann and Creutzfeldt, 2011) suggested that female cerebral organization of language may be different, sometimes giving women an advantage over males in performing linguistic tasks that may be critical in SLI. These include verbal memory (Sundermann et al., 2016) and speech production (Hyde and Linn, 1988). Such male underrepresentation in the study causes even more concern since the majority of interpreters employed at international organizations are female (Fabbro and Gran, 1994). More recent survey results show that among the members of the International Association of Conference Interpreters (AIIC) about three quarters are female (Buck and Luccarelli, 2005), which suggests a possible female advantage in this SLI that may be due to cognitive factors. However unlikely, the enhanced connectivity might not replicate in a sample consisting mostly or fully of males.

The authors also reported a statistically significant (p = 0.032) positive partial correlation (r = 0.576), after controlling for age, between the cumulative number of training hours across lifespan and mean functional connectivity between the ROIs. However, this correlation should be taken with caution for several reasons. First, it is hard for an interpreter to report exactly how many hours he or she has worked unless they keep a record of the time spent in the booth throughout their entire career. This means that an estimate of the cumulative number of working (or training) hours can only be obtained by multiplying the duration of an SI's career in years by an expected number of working hours per year. Such an estimate would be quite noisy, which in a small sample may lead to spurious results. Second, although the significance criterion (p < 0.05) was met using a parametric method, with a relatively small number of participants it is hard to ensure that the assumptions of the Pearson product moment correlation have not been violated. Hence, a more robust hypothesis testing method would be more appropriate than the conventional asymptotic normal distribution technique. Indeed, according to a bootstrapped analysis of the data with 50,000 samples, the 95% confidence interval for the correlation between the cumulative duration of training and functional connectivity was very broad (−0.097, 0.930), which indicates a non-significant result. Finally, the small sample size for this correlation does not guarantee a high level of statistical power (which our estimate showed to be around 0.65).

In future research, it would be interesting to measure the functional connectivity in SI students before and after their training programs in conference interpreting and with a specific focus on possible gender effects. Such a longitudinal study could reveal neural correlates of performance at entrance and final exams and ultimately help resolve the nature vs. nurture debate in the field of interpretation studies.

## Author contributions

RK conceptualized the paper, drafted the manuscript, performed the data analyses and interpretation of the results. AO participated in the literature review for the manuscript and interpretation of the results as well as reviewed the manuscript. The authors agreed to the final version of the manuscript.

### Conflict of interest statement

The authors declare that the research was conducted in the absence of any commercial or financial relationships that could be construed as a potential conflict of interest.
